# Patient-reported experience measures in patients undergoing navigated transcranial magnetic stimulation (nTMS): the introduction of nTMS-PREMs

**DOI:** 10.1007/s00701-020-04268-y

**Published:** 2020-02-25

**Authors:** Sabina Patel, Prajwal Ghimire, José Pedro Lavrador, Josephine Jung, Richard Gullan, Keyoumars Ashkan, Ranjeev Bhangoo, Francesco Vergani

**Affiliations:** Department of Neurosurgery, King’s College Hospital Foundation Trust, London, UK

**Keywords:** PREMs, nTMS, Unmet needs, Quality of care

## Abstract

**Background:**

Patient-reported experience measures (PREMs) are a unique measure of experience of patients which can help address the quality of care of the patients.

**Objective:**

Our aim of the study is to collect quality of care outcomes with our newly navigated transcranial magnetic stimulation patient-reported experience measure (nTMS-PREMs) questionnaire among neurosurgical patients undergoing nTMS.

**Methods:**

A single-centre prospective nTMS-PREMs 19-item questionnaire study was performed between February 2018 and December 2018 on patient referred for nTMS at our hospital. The Data was analysed using Likert scale, linear and logistic regression using statistical software (STATA 13.0®).

**Results:**

Fifty patient questionnaires were collected (30 males, 20 females, mean age of 47.6 ± 2.1 years) among which 74% of patients underwent both motor and language mapping with a mean duration of 103.3 ± 5.1 min. An overall positive response was noted from the results of the questionnaire, tiredness and anxiety being the common effects noted. Patients with the left-sided disease appreciated more the conditions provided in our laboratory (Q4, *p* = 0.040) and increasing age was related to less confidence and trust (Q6, *p* = 0.038) in the staff performing the exam. Younger patients tolerated nTMS better than older patients (> 65 years). PubMed literature search resulted in no relevant articles on the use of PREMs in nTMS patients.

**Conclusion:**

nTMS is a well-tolerated non-invasive tool and nTMS-PREMS provides a promising role in identifying the unmet needs of the patients and improving the quality of their care.

**Electronic supplementary material:**

The online version of this article (10.1007/s00701-020-04268-y) contains supplementary material, which is available to authorized users.

## Introduction

Navigated transcranial magnetic stimulation (nTMS) has become in recent years a commonly used tool in the pre-operative planning for neurosurgical patients [8]. nTMS is now being widely used as an important pre-operative tool for motor and speech mapping in eloquent glioma surgery (awake/asleep), brain metastasis, cavernoma, and vascular malformations [5, 7, 14, 15, 17, 20, 23, 25] and is a useful adjunct to impact the surgical indications, guide the surgical approach, and estimate and reduce the risk of inducing post-surgical deficits [26, 27]. It has further been highlighted in recent studies that the preoperative nTMS motor mapping/speech mapping provides improved outcome in patients with glioblastoma [24] and is also useful in language-eloquent tumour surgery in awake-ineligible patients [25]. While a safe and non-invasive way to map the cerebral cortex, nTMS can be associated with anxiety or unpleasant experience [14] or discomfort [28] and can be time-consuming, with motor mapping requiring up to 90 min and language mapping requiring up to 120 min [8]. To date, little is known about the overall patients’ experience during nTMS, with particular regard to how well the procedure is tolerated and what additional information it gives to patients in their overall healthcare experience. This is of particular relevance, since patients’ collaboration and understanding of nTMS are crucial for the success of the examination.

Patient-reported experience measures (PREMs) are increasingly used to assess the effectiveness of clinical care on a particular patient population (for example, Picker Patient Experience Questionnaire (PPE-15), Patient Experience Questionnaire (PEQ), Scottish Inpatient Patient Experience Survey (SIPES), Norwegian Patient Experience Questionnaire (NORPEQ), In-Patient Experiences of Health Care (I-PAHC)) [1–4, 11]. In neurosurgery, studies to evaluate patients’ experience and satisfaction have been performed, for example, in patients undergoing awake craniotomy [10, 18, 30]. However, no specific PREMs have been reported for the use of nTMS. In the present paper, we aim to document the first patient-reported experience measure for nTMS in neurosurgery (nTMS-PREMs).

## Material and methods

A prospective study was carried out between January and December 2018 at our hospital. All patients (age ≥ 18 years old) who were referred for TMS at our Institution were involved. Patients were referred for TMS after multidisciplinary team (MDT) discussions when a lesion in a presumed eloquent brain was identified. All patients were instructed about TMS in person at the time of the first outpatient meeting in the neuro-oncology clinic and again on the day of the exam. In addition, patients and their families were supplied with a booklet explaining the details of the exam. TMS was performed as an outpatient procedure. A T1 post-contrast MRI for navigation purposes was acquired before or on the day of the exam. Patients who had the MRI on the same day of TMS had only a T1 post-gadolinium MRI sequence for navigation purposes and were given a break of approximately 1 h before proceeding to TMS. All patients underwent either motor mapping alone or a combination of motor and language mapping. In the latter case, motor and language mapping were performed on the same day as a continuous exam. Motor mapping was performed with single pulse stimulation at 105% of the resting motor threshold (RMT) while language mapping was performed with repetitive stimulation at 100% of the RMT. Two authors (S.P. and J.P.L.) performed all the mappings. After the mapping, the results were explained and discussed with patients, with particular regard to the relationship between positive responses and tumour location. Details of the nTMS protocol at our institution have been previously reported [8].

After the exam, all patients were invited to anonymously fill an ad hoc nTMS-PREMs questionnaire (Supplementary Material 1). The questionnaire was designed with reference to the Royal College of Surgeons (RCS) of England survey/audit indications with domains covering quality of the information provided, facilities of the laboratory, the test/examination, staff evaluation, and discharge information [19]. The questionnaire evaluated the following domains of the TMS experience: background (the quality of the information provided before the exam, 3 questions); laboratory (the facilities of the TMS laboratory, 2 questions); staff (evaluating the examiners performing the procedure, 3 questions); exam (the experience during the exam itself, 5 questions); discharge information (the quality and accuracy of the information provided at discharge, 4 questions); and the overall rating of the nTMS experience (2 questions). One question within the “exam” section was aimed only at patients who had language mapping alongside the motor mapping. Each question was rated as a scale from 1 (very poor experience) to 5 (excellent experience). Each question was addressed individually as well as part of the 6 domains where the combined score (combined results of all questions within that domain) was determined. In order to provide a better global evaluation of each question and domain, the responses were also combined as follows: poor, ratings 1 and 2; neutral, rating 3; and good, ratings 4 and 5.

The following variables were analysed against each question and domain: age, gender, duration of the exam, and type of mapping (motor versus motor and language). The laterality of the disease, location of the disease, and RMT ratio (RMT disease side/RMT healthy side)—pathological (90% < RMT < 110%) versus non-pathological (all other values)—were assessed against each question in the “exam” domain. Linear regression was used for age and duration of the exam. Logistic regression was used for gender, laterality of the disease, RMT ratio, and type of mapping. Multinomial logistic regression was used for location. The combined score analysis was performed using ordered logistic regression analysis. A multivariate analysis was performed for the 6 domains. An adjustment for age, gender, duration, and type of monitoring was used for every domain and laterality and RMT ratio for the “exam” domain. A subgroup analysis for the older population was performed. *p* values < 0.05 were considered significant for all the performed analysis. Statistical software (STATA 13.0®) was used for all the performed analysis. All the analyses were performed after the study was completed.

In addition, a literature search was performed in PubMed to look for previous papers reporting PREMS related to TMS. We used MeSH terms related to nTMS and PREMS. There were no articles in the search that was dedicated to specific experience measures in nTMS. The study was registered in the local audit registry as a service improvement project approved by the Neurosurgical Department. Informed consent was taken with all the patients in the study for nTMS mapping. The pre-operative mapping was applied following the standard nTMS mapping protocol published previously [8].

## Results

Patients were referred from both the neuro-oncology and neurovascular services with a total of 50 questionnaires completed (30 males, 20 females, mean age of 47.6 ± 2.1 years)—2 patients did not return the questionnaires. The vast majority had an underlying brain tumour—46 (92.0%). The preferential location was on the left side (29 patients, 60.4%) with the frontal (22 cases, 48.9%) and temporal (13 cases, 28.9%) lobes being more commonly affected. Twenty-one patients (46.67%) had a pathological RMT ratio (mean RMT ratio, 1.0 ± 0.02). Seventy-four percent of patients underwent both motor and language mapping with a mean duration of 103.3 ± 5.1 min (motor only, 85.8 ± 6.1 min; motor and speech, 106.9 ± 5.9 min; *p* = 0.02, *t* test) (Table 1).

**Table 1:** Demographic characteristics of the study population

	nTMS (*n* = 50)
Age	47.6 ± 2.1
Gender	
Male	30 (60.0%)
Female	20 (40.0%)
Pathology	
Oncology	46 (92.0%)
Vascular	4 (8.0%)
Type of mapping	
Motor only	13 (26.0%)
Speech and motor	37 (74.0%)
Laterality	
R ight	19 (39.6%)
Left	29 (60.4%)
Location (lobe)	
Insula	3 (6.7%)
Frontal	22 (48.9%)
Parietal	7 (15.6%)
Temporal	13 (28.9%)
RMT	
Ipsilateral to the disease (%)	41.1 ± 1.7
Contralateral to the disease (%)	40.5 ± 1.5
RMT ratio	1.0 ± 0.02
Pathological RMT (patients)	21 (46.64%)
Duration (min)	103.3 ± 5.1
Motor only	85.8 ± 6.1
Speech and motor	106.9 ± 5.9

A breakdown of responses to the questionnaire is reported in Table 2. In general, patients reported a positive experience with nTMS, with 94% positive responses for the overall nTMS experience. Seven hundred ninety-two (85%) of the responses provided about our service were rated as good, and 648 (69%) rated with the maximum score. Taking into account the individual domains of the questionnaire, 89% of patients reported a positive score for the “background” domain, with a good understanding of TMS and availability of information prior to TMS taking place with only 7% rating this section poorly. The combined score for the “laboratory” domain was good in 85% of responses with 75% reporting no technical problems during the exam. The “staff” section had the highest positive rating, with 95% good responses, indicating confidence in the staff performing the investigation (95%), presence of knowledge (92%), and support from staff (96%). The “exam” domain had the lowest positive rating (70% as good) with a poor experience due to anxiety and pain reported by 26% and 24% of patients, respectively. In addition, 16% of patients reported tiredness during/after the exam and difficulties in concentration. Reassuringly, 94% of patients felt the exam duration met their expectations and was acceptable. The quality of the “discharge information” provided was well rated by the patients (84% rated as good) with 88% of patients feeling they had a better understanding of the relationship between their lesion and the eloquent areas of the brain and 70% of patients clearly understood the importance of nTMS in the context of the surgical treatment they were about to receive.

**Table 2:** Results of the nTMS-PREMs questionnaire

		Rating						Classification			Overall		
	Questions	1	2	3	4	5	NA	Poor	Neutral	Good	Poor	Neutral	Good
Intro	1. How much information has been given to you about TMS?	2 (4%)	1 (2%)	2 (4%)	11 (22%)	34 (68%)	–	3 (6%)	2 (4%)	45 (90%)	11 (7%)	5 (3%)	133 (89%)
	2. How much understanding you had about the importance and role of TMS in the treatment of your disease?	1 (2%)	3 (6%)	2 (4%)	10 (20%)	34 (68%)	–	4 (8%)	2 (4%)	44 (88%)			
	3. Have you been given enough time and privacy to discuss TMS?	2 (4%)	2 (4%)	1 (2%)	6 (12%)	38 (76%)	1 (2%)	4 (8%)	1 (2%)	44 (88%)			
Lab	4. Was the laboratory quiet so you could concentrate and collaborate during the exam?	1 (2%)	–	2 (4%)	7 (14%)	40 (80%)	–	1 (2%)	2 (4%)	47 (94%)	7 (7%)	6 (6%)	85 (85%)
	5. Was there any technical problem during the course of the exam?	5 (10%)	1 (2%)	4 (8%)	12 (24%)	26 (52%)	2 (4%)	6 (12%)	4 (8%)	38 (76%)			
Staff	6. Did you have confidence and trust in the staff performing the exam?	1 (2%)	–	–	4 (8%)	45 (90%)	–	1 (2%)	–	49 (98%)	6 (4%)	1 (0.6%)	143 (95%)
	7. Did you recognize knowledge and experience about your condition in the members of the staff?	2 (4%)	1 (2%)	1 (2%)	5 (10%)	41 (82%)	–	3 /6%)	1 (2%)	46 (92%)			
	8. Did the members of staff provide you support during the exam?	2 (4%)	–	–	4 (8%)	44 (88%)	–	2 (4%)	–	48 (96%)			
Exam	9. Do you think the exam duration was in line with your expectations and acceptable?	1 (2%)	–	2 (4%)	7 (14%)	40 (80%)	–	1 (2%)	2 (4%)	47 (94%)	40 (17%)	30 (13%)	165 (70%)
	10. Did you feel tired during the exam? (1 very tired, 5 not tired at all)	3 (6%)	5 (10%)	8 (16%)	15 (30%)	18 (36%)	1 (2%)	8 (16%)	8 (16%)	33 (66%)			
	11. Were you able to concentrate during the exam? (Speech mapping only)^*^	4 (11%)	2 (5%)	6 (16%)	8 (22%)	17 (46%)	–	6 (16%)	6 (16%)	25 (68%)			
	12. Were you anxious during the exam? (1 very anxious, 5 not anxious at all)	12 (24%)	1 (2%)	6 (12%)	4 (8%)	26 (52%)	1 (2%)	13 (26%)	6 (12%)	30 (60%)			
	13. Did you feel pain during the exam? (1 very painful, 5 not painful at all)	6 (12%)	6 (12%)	8 (16%)	8 (16%)	22 (44%)	–	12 (24%)	8 (16%)	30 (60%)			
Discharge	14. Were the explanations of the results important for your understanding of the operation?	2 (4%)	1 (2%)	4 (8%)	8 (16%)	32 (64%)	3 (6%)	3 (6%)	4 (8%)	35 (70%)	11 (6%)	9 (4%)	167 (84%)
	15. Did TMS help you to understand the relationship between the lesion and the brain areas that control function?	3 (6%)	–	2 (4%)	9 (18%)	35 (70%)	1 (2%)	3 (6%)	2 (4%)	44 (88%)			
	16. Have your concerns been supported and answered by the team staff?	2 (4%)	–	1 (2%)	7 (14%)	38 (76%)	2 (4%)	2 (4%)	1 (2%)	45 (90%)			
	17. Has the post-exam instructions / follow-up plan been explained to you satisfactorily at the end of the exam?	3 (6%)	–	2 (4%)	10 (20%)	33 (66%)	2 (4%)	3 (6%)	2 (4%)	43 (86%)			
End	18. Do you think after completing this exam that TMS is an important part of your treatment plan?	1 (2%)	–	1 (2%)	5 (10%)	42 (84%)	1 (2%)	1 (2%)	1 (2%)	47 (94%)	3 (3%)	2 (2%)	94 (94%)
	19. How would you rate your overall TMS experience? Would you have TMS mapping again?	2 (4%)	–	1 (2%)	4 (8%)	43 (86%)	–	2 (4%)	1 (2%)	47 (94%)			
Total		55 (7%)	23 (2%)	53 (6%)	144 (15%)	648 (69%)	14 (1%)	78 (9%)	53 (6%)	792 (85%)			

13 patients were excluded—motor mapping only

The significant results from the univariate statistical analysis are reported in Table 3 (the complete table of results is provided in Supplementary Table 2). An increased duration of the exam was related to increased pain felt during the exam (Q13, *p* = 0.004) and a poorer understanding of the significance of the results for the surgical treatment (Q14, *p* = 0.031). Increasing age was related to less confidence and trust (Q6, *p* = 0.038) and less ability to recognize knowledge and experience (Q7, *p* = 0.003) in the staff performing the exam (Table 3).

**Table 3:** Summary of the positive findings in the univariate analysis for nTMS-PREMs questionnaire

	Coef.	95% confidence interval	*p* value
Q4. Was the laboratory quiet so you could concentrate and collaborate during the exam?			
Laterality (left side)	1.400	[0.064–2.737]	0.040
Q6. Did you have confidence and trust in the staff performing the exam?			
Age	− 7.055	[− 13.708 to − 0.408]	0.038
Q7. Did you recognize knowledge and experience about your condition in the members of the staff?			
Age	− 6.313	[− 10.411 to − 2.214]	0.003
Q13. Did you feel pain during the exam? (1 very painful, 5 not painful at all)			
Duration	− 9.501	[− 15.793 to − 3.209]	0.004
Q14. Were the explanations of the results important for your understanding of the operation?			
Duration	− 9.023	[− 17.189 to − 0.857]	0.031

The univariate analysis was also repeated against each domain of the questionnaire. This revealed that longer exams were related to a poorer rate of the laboratory conditions (*p* = 0.017). Increasing age was related to a poorer rate of staff performance (*p* = 0.001). Concerning the exam, the patients who had only motor mapping had a better experience than the ones who had both motor and language mapping (*p* = 0.020). Patients who had frontal lesions and longer mapping reported worse experiences (*p* = 0.028 and *p* = 0.003). The quality of the information provided at discharge was better in patients who had motor mapping only (*p* = 0.001) and worse in patients who had longer mappings (*p* = 0.002). None of the studied variables seemed to influence the way patients rated their overall experience of nTMS (all *p* > 0.05) (Table 4).

**Table 4:** Univariate analysis for the 6 domains of the nTMS-PREMs questionnaire

	Coef.	95% Confidence Interval	*p* value
Introduction			
Age	− 0.015	[− 0.037–0.014]	0.378
Gender	− 0.324	[− 1.042–0.394]	0.377
Laterality	0.424	[− 0.288–1.135]	0.244
Location			
Frontal	0.589	[0.070–1.108]	0.026
Parietal	0.611	[− 0.048–1.271]	0.069
Temporal	0.438	[− 0.091–0.966]	0.104
RMT ratio	0.240	[− 0.479–0.960]	0.512
Duration	−0.011	[− 0.024–0.003]	0.121
Type of mapping	0.901	[− 0.0003–1.802]	0.050
Laboratory			
Age	0.003	[− 0.027–0.032]	0.866
Gender	− 0.832	[− 1.730–0.066]	0.069
Laterality	1.308	[0.428–2.188]	0.004
Location			
Frontal	0.074	[− 0.600–0.748]	0.830
Parietal	0.459	[− 0.472–1.390]	0.334
Temporal	0.190	[− 0.537–0.916]	0.609
RMT ratio	− 0.045	[− 0.900–0.810]	0.917
Duration	− 0.019	[− 0.035 to − 0.003]	0.017
Type of mapping	− 0.390	[− 1.304–0.524]	0.402
Staff			
Age	− 0.076	[− .122 to − 0.030]	0.001
Gender	− 0.806	[− 1.875–0.262]	0.139
Laterality	0.074	[− 0.889–1.037]	0.880
Location			
Frontal	− 13.414	[− 1510.328–1483.499]	0.986
Parietal	− 13.303	[− 1510.218–1483.61]	0.986
Temporal	− 13.037	[− 1509.95–1483.877]	0.986
RMT ratio	0.351	[− 0.612–1.315]	0.475
Duration	− 0.012	[− 0.028–0.004]	0.155
Type of mapping	1.259	[−0.251–2.768]	0.102
Exam			
Age	− 0.008	[− 0.025–0.009]	0.331
Gender	− 0.428	[− 0.929–0.073]	0.094
Laterality	0.166	[− 0.333–0.665]	0.513
Location			
Frontal	− 1.375	[− 2.602 to − 0.147]	0.028
Parietal	− 0.985	[− 2.211–0.295]	0.134
Temporal	− 1.162	[− 2.395–0.070]	0.065
RMT ratio	− 0.027	[− 0.530–0.476]	0.916
Duration	− 0.014	[− 0.023 to − 0.005]	0.003
Type of mapping	0.748	[0.118–1.378]	0.020
Discharge information			
Age	−0.17	[−0.040–0.006]	0.139
Gender	−0.423	[−1.067–0.221]	0.198
Laterality	0.407	[−0.231–1.044]	0.212
Location			
Frontal	− 0.302	[− 1.060–0.457]	0.436
Parietal	0.201	[− 0.741–1.143]	0.676
Temporal	− 0.189	[− 0.971–0.592]	0.635
RMT ratio	− 0.080	[− 0.718–0.557]	0.804
Duration	− 0.020	[− 0.033 to − 0.008]	0.002
Type of mapping	2.037	[0.819–3.254]	0.001
Overall			
Age	− 0.013	[− 0.057–0.030]	0.542
Gender	− 0.674	[− 1.908–0.560]	0.285
Laterality	0.185	[− 0.960–1.331]	0.751
Location			
Frontal	0.216	[− 0.583–1.015]	0.597
Parietal	0.230	[− 0.733–1.193]	0.639
Temporal	0.392	[− 0.551–1.335]	0.415
RMT ratio	− 0.083	[− 1.221–1.054]	0.886
Duration	− 0.010	[− 0.033–0.012]	0.372
Type of mapping	1.708	[− 0.3780–3.794]	0.109

The multivariate analysis confirmed age (*p* = 0.01) and duration of the exam (*p* = 0.05) as two major factors influencing the rating of the TMS exam. Increasing age was related to a poorer rating in almost *all domains* with exception of the laboratory conditions and increased duration of the exam was related to the poor rating of laboratory conditions (*p* = 0.03) and worse discharge information (*p* = 0.003) (Table 5 and Supplemental Table 3)

**Table 5:** Positive findings of multivariate analysis for the 6 domains of the nTMS-PREMs questionnaire

	Coef.	95% confidence interval	*p* value
Introduction			
Age	− 0.034	[− 0.064 to − 0.003]	0.030
Laboratory			
Duration			
	− 0.0181	[− 0.0.4 to − 0.0014]	0.034
Staff			
Age			
	− 0.351	[− 0.669 to − 0.0329]	0.031
Exam			
Age			
	− 0.028	[− 0.050 to − 0.007]	0.01
Discharge information			
Age	− 0.044	[− 0.071 to − 0.017]	0.002
Duration	− 0.020	[− 0.034 to − 0.007]	0.003
Overall			
Age			
	− 0.089	[− 0.159 to − 0.019]	0.013

## Discussion

PREMs play an important role in evaluating the experience of patients to an intervention that leads to the evaluation of patients’ satisfaction and is useful for research, quality improvement projects, clinician performance evaluation, audit, and economic evaluation [1, 11]. In recent years, generic- and intervention-specific PREMs have been developed and used in different areas of clinical medicine and surgical practice, stressing the relevance of patient satisfaction to a patient-centred approach in modern medicine [1, 3, 4, 11]. Well-established surgical PREMs have already been developed for specific surgical interventions, like hernia repair or hip replacement and these have been approved for use in the UK and worldwide [2]. In neurosurgery, intervention-specific PREMs will act as an important indicator of the quality of care of neurosurgical patients and enhance research, quality governance, and economic evaluation/cost-effectiveness of the overall treatment/surgical decision-making and execution.

Over the last decade, nTMS has been used by neurosurgeons to map functional areas of the brain for surgical planning in neuro-oncology [5, 22], AVM resection [7, 9, 16], and epilepsy [13]. A successful nTMS depends largely on patient cooperation, with a long duration of the exam and fatigue or discomfort potentially affecting the results of the mapping. PREMs specifically designed to assess the nTMS experience can give a valuable insight into patient counselling, experience during the procedure, side effects, duration of the test, and how the results of the mapping are understood and perceived by patients. The information provided can in turn be used as a feedback for the nTMS team to allow self-assessment and service improvement. Our literature search through PubMed showed that there are currently no PREMs specifically designed for TMS. Previous papers looked at patient experience during repetitive TMS for therapeutic application [12]. Singh et al. [28] looked at experience and attitudes in patients undergoing repetitive TMS for psychiatric conditions in North India over a 3-month period. They noted an overall positive experience in the context of repetitive TMS for treatment of depression, with a majority agreeing they would recommend TMS to others. A similar result was reported by an earlier study carried out in Tasmania in 2001, again in the context of rTMS for depression [6]. In neurosurgery, recent studies [14, 23, 28, 29] focused on the occurrence of pain, discomfort, and seizures during pre-surgical brain mapping. The authors concluded that nTMS is safe and generally well tolerated. Despite giving useful information on the safety and tolerability of nTMS, this study did not report PREMs addressing the whole patient experience.

In the present paper, we report the first specific nTMS-PREMs. To this effect, we designed a patient questionnaire following the indications of the Royal College of Surgeon in auditing patient experience and satisfaction and the published literature review on commonly developed PREMs [1, 19]. As a result, six domains were identified and evaluated: background information, laboratory, staff, exam experience, discharge information, and overall rating of the nTMS experience. Our results show that nTMS is largely well-tolerated and patients have an overall good experience with nTMS at different time points of the mapping. In all domains, there was a majority of positive responses, with 94% of positive responses for the overall nTMS assessment. However, approximately one in four patients reported the occurrence of anxiety and pain during the exam. Both univariate analysis and multivariate analysis confirmed the age and duration of exam as two major factors that influence patients’ experience during the TMS procedure. The univariate analysis clarified that longer duration of mapping and language mapping are related to a poorer experience. The poorer experience with language mapping can be explained on the basis of the longer duration of the test and on the discomfort/pain induced by repetitive stimulation involving the temporalis muscle [27–29]. Similarly, an increased duration of the exam was related to a poorer understanding of the discharge information, with particular regard to the role of TMS in planning the surgical treatment. We speculate that this is due to the difficulty of patients to maintain attention and retain information due to fatigue after a long mapping session. Elderly patients tended to give a poorer assessment of the staff performing nTMS. This result may be explained on the basis that only very few negative responses were reported concerning the staff (with a possible influence on the statistical result). However, we acknowledge that nTMS was performed by relatively junior members within the neuro-oncology team and therefore, we cannot exclude that a perceived “generational gap” between patients and healthcare professionals may have played a role [21, 31].

The information gathered from the PREMs recorded in this study can be used to improve clinical practice. The poor experience reported with long duration of mapping can be mitigated by ensuring patients can take appropriate rest. It has now become customary at our institution to offer patients a break in between motor and language mapping, so that they can be more relaxed and focused during the language testing. In addition, on top of providing patients with a booklet containing all the information about nTMS before the testing, we make sure to spend enough time to explain the results of mapping, showing patients their nTMS-generated maps. After nTMS, the majority of patients reported a good understanding of the relationship between the tumour and functional areas of the brain. This is relevant, as nTMS results can be used for better patient counselling at the time of consenting for an operation. It is now a routine at our institution to include nTMS-generated maps, when available, as part of the information presented to patients and relatives when describing the challenges and risks of a specific neurosurgical intervention in a highly functional area.

## Limitations

This is a single-centre pilot study in a limited number of patients. Further multicentric, international studies are warranted to further validate our questionnaire and confirm the generalisability of our results across different countries and patient populations. Our pilot, single-centre experience will serve as the necessary basis for such studies.

## Conclusion

nTMS is an overall well-tolerated procedure, with positive feedback reported by the vast majority of patients. Poorer experience has been identified in elderly patients and in patients undergoing long mapping, with particular regard to pain and understanding of nTMS results. This information can help in tailoring the nTMS experience to individual patients’ needs.

## Electronic supplementary material

ESM 1 (PDF 84 kb)

ESM 2 (DOCX 15 kb)

ESM 3 (DOCX 16 kb)
