# Supplementary material for: Patient-reported experience measures in patients undergoing navigated transcranial magnetic stimulation (nTMS): the introduction of nTMS-PREMs
Source: Acta Neurochir (Wien). 2020 Feb 25;162(7):1673–81. doi: 10.1007/s00701-020-04268-y (PMC7295840; doi:10.1007/s00701-020-04268-y)
Supplement: Supplementary file 1 — (PDF 84 kb) [file 701_2020_4268_MOESM1_ESM.pdf]

XXX

Transcranial Magnetic Stimulation – Patient Reported Outcome Measurement (PREM-TMS)

This questionnaire is about your Transcranial Magnetic Stimulation session and your overall experience during its course. Your responses will be a valuable tool in order to improve the care we provide to our patients in the Neurosurgical Unit at XXX Hospital.

This is a voluntary survey. If you would prefer not to take part in it, you don't need to provide any reason. If you wish to participate, we would strongly appreciate your contribution.

Demographic and clinical information will be collected to help staff members to interpret the results and will be treated confidentially.

Please complete the questionnaire answering as many questions as you can but don't feel obliged to respond to all of them if you don't wish to do so.

Note that the numeric responses represent a scale from 1 being very negative to 5 being very positive and 3 being neutral.

After completing this questionnaire please hand it back to the medical staff.

For Staff use only

Demographic Data

Hospital No.:

Age: Gender:

Location of the Lesion: ◯ Left ◯ Right Lobe:

Stimulation Findings: RMT Left UL Right UL

Left LL Right LL

Language

Duration of the Exam [min]:

Patient

Background Information

How much information has been given to you about TMS?

1	2	3	4	5
not at all				very much

How much understanding you had about the importance and role of TMS in the treatment of your disease?

1	2	3	4	5
not at all				very much

Have you been given enough time and privacy to discuss TMS?

1	2	3	4	5
not at all				very much

Laboratory

Was the laboratory quiet so you could concentrate and collaborate during the exam?

1	2	3	4	5
not at all				very much

Have you noted technical problems during the course of the exam?

1	2	3	4	5
very poor				very much

Staff

Did you have confidence and trust in the staff performing the exam?

1 2 3 4 5

not at all very much

Did you recognize knowledge and experience about your condition in the members of the staff?

1 2 3 4 5

not at all very much

Did the members of staff provide you support during the exam?

1 2 3 4 5

not at all very good

Exam

Do you think the exam duration was in line with your expectations and acceptable?

1 2 3 4 5

not at all very good

Did you feel tired during the exam? (1 very tired, 5 not tired at all)

1 2 3 4 5

very tired not tired at all

Were you able to concentrate during the exam? (Speech mapping only)

1 2 3 4 5

not at all very much

Were you anxious during the exam? (1 very anxious, 5 not anxious at all)

1 2 3 4 5

very anxious not anxious at all

Did you feel pain during the exam? (1 very painful, 5 not painful at all)

1 2 3 4 5

very painful not painful at all

Post-Exam Feedback / Discharge Information

Have the main results of the exam been explained to you?

1 2 3 4 5

not at all very much

Did TMS help you to understand the relationship between the lesion and the brain areas that control function?

1 2 3 4 5

not at all very much

Have your concerns been supported and answered by the team staff?

1 2 3 4 5

not all very much

Has the post-exam instructions / follow-up plan been explained to you satisfactorily at the end of the exam?

1 2 3 4 5

not at all very much

Overall

Do you think after completing this exam that TMS is an important part of your treatment plan?

1	2	3	4	5
very poor				very much

How would you rate your overall TMS experience?

1	2	3	4	5
very poor				very good

Please provide any comment/suggestion that can help us to improve our service:

Your feedback is greatly appreciated.

Thank you
