# Supplementary material for: Patient-reported experience measures in patients undergoing navigated transcranial magnetic stimulation (nTMS): the introduction of nTMS-PREMs
Source: Acta Neurochir (Wien). 2020 Feb 25;162(7):1673–81. doi: 10.1007/s00701-020-04268-y (PMC7295840; doi:10.1007/s00701-020-04268-y)
Supplement: Supplementary file 2 — (DOCX 15 kb) [file 701_2020_4268_MOESM2_ESM.docx]

|  | **Q1** | **Q2** | **Q3** | **Q4** | **Q5** | **Q6** | **Q7** | **Q8** | **Q9** | **Q10** | **Q11** | **Q12** | **Q13** | **Q14** | **Q15** | **Q16** | **Q17** | **Q18** | **Q19** |
| --- | --- | --- | --- | --- | --- | --- | --- | --- | --- | --- | --- | --- | --- | --- | --- | --- | --- | --- | --- |
| **Age** | 0.247 | 0.313 | 0.123 | 0.174 | 0.313 | 0.038 | 0.003 | 0.111 | 0.265 | 0.779 | 0.400 | 0.615 | 0.662 | 0.351 | 0.080 | 0.086 | 0.147 | 0.088 | 0.194 |
| **Gender** | 0.324 | 0.173 | 0.573 | 0.151 | 0.180 | 0.377 | 0.178 | 0.943 | 0.694 | 0.400 | 0.482 | 0.112 | 0.936 | 0.140 | 0.736 | 0.257 | 0.521 | 0.331 | 0.283 |
| **Laterality** | 0.279 | 0.693 | 0.380 | 0.040 | 0.178 | 0.424 | 0.385 | 0.929 | 0.675 | 0.438 | 0.545 | 0.664 | 0.523 | 0.359 | 0.903 | 0.652 | 0.689 | 0.313 | 0.876 |
| **Location**  Frontal  Parietal Temporal | 0.066  0.215  0.212 | 0.182  0.267  0.409 | 0.510  0.367  0.469 | 0.993  0.994  0.994 | 0.448  0.183  0.412 | 0.993  0.993  0.993 | 0.991  0.991  0.991 | 0.994  0.994  0.994 | 0.994  0.994  0.994 | 0.211  0.652  0.225 | 0.811  0.896  0.947 | 0.995  0.996  0.995 | 0.995  0.995  0.995 | 0.910  0.404 0.740 | 0.993  0.993  0.993 | 0.989  0.989  0.989 | 1.000  0.529  0.942 | 0.221  0.363  0.990 | -  -  - |
| **RMT Ratio** | 0.361 | 0.905 | 1.000 | 0.696 | 0.820 | 0.459 | 0.903 | 0.284 | 1.000 | 0.301 | 0.526 | 0.760 | 0.910 | 0.825 | 0.858 | 0.302 | 0.932 | 0.916 | 0.415 |
| **Duration** | 0.514 | 0.851 | 0.451 | 0.386 | 0.062 | 0.835 | 0.485 | 0.835 | 0.594 | 0.485 | 0.830 | 0.225 | 0.004 | 0.031 | 0.051 | 0.116 | 0.188 | 0.436 | 0.089 |
| **Type** | 0.150 | 0.209 | 0.268 | 0.693 | 0.567 | 0.993 | 0.912 | 0.993 | 0.238 | 0.744 | - | 0.272 | 0.080 | 0.132 | 0.156 | 0.992 | 0.150 | 0.401 | 0.993 |

**Supplemental table 2 (*for Table 3*): P values as per each question in the nTMS-PREMs questionnaire**
