# Supplementary material for: Patient-reported experience measures in patients undergoing navigated transcranial magnetic stimulation (nTMS): the introduction of nTMS-PREMs
Source: Acta Neurochir (Wien). 2020 Feb 25;162(7):1673–81. doi: 10.1007/s00701-020-04268-y (PMC7295840; doi:10.1007/s00701-020-04268-y)
Supplement: Supplementary file 3 — (DOCX 16 kb) [file 701_2020_4268_MOESM3_ESM.docx]

| **Introduction** | | | |
| --- | --- | --- | --- |
|  | **Coef.** | **95% Confidence Interval** | **p value** |
| **Age** | -0.034 | [-0.064 - -0.003] | 0.030 |
| **Gender** | -0.1222 | [-1.099 – 0.854] | 0.806 |
| **Duration** | -0.0089 | [-0.0232 – 0.0054] | 0.224 |
| **Type of Mapping** | 1.297 | [-0.300 – 2.895] | 0.112 |
| **Laboratory** | | | |
|  | **Coef.** | **95% Confidence Interval** | **p value** |
| **Age** | -0.0122 | [-0.046 – 0.022] | 0.486 |
| **Gender** | -1.117 | [-2.530 – 0.294] | 0.121 |
| **Duration** | -0.0181 | [-0.0.4 - -0.0014] | 0.034 |
| **Type of Mapping** | -0.516 | [-2.063 – 1.029] | 0.512 |
| **Staff** | | | |
|  | **Coef.** | **95% Confidence Interval** | **p value** |
| **Age** | -0.351 | [-0.669 - -0.0329] | 0.031 |
| **Gender** | -16.652 | [-3787.4 – 3754.1] | 0.993 |
| **Duration** | -0.016 | [-0.039 – 0.007] | 0.176 |
| **Type of Mapping** | -2.286 | [-5.405 – 0.833] | 0.151 |
| **Exam** | | | |
|  | **Coef.** | **95% Confidence Interval** | **p value** |
| **Age** | -0.028 | [-0.050 - -0.007] | 0.01 |
| **Gender** | -0.684 | [-1.450 – 0.083] | 0.081 |
| **Laterality** | 0.164 | [-0.509 – 0.837] | 0.633 |
| **RMT Ratio** | -0.317 | [-0.965 – 0.330] | 0.337 |
| **Duration** | -0.010 | [-0.020 - 0.0003] | 0.058 |
| **Type of Mapping** | 0.792 | [-0.174 – 1.759] | 0.108 |
| **Discharge Information** | | | |
|  | **Coef.** | **95% Confidence Interval** | **p value** |
| **Age** | -0.044 | [-0.071 - -0.017] | 0.002 |
| **Gender** | -.0804 | [-1.769 – 0.161] | 0.102 |
| **Duration** | -0.020 | [-0.034 - -0.007] | 0.003 |
| **Type of Mapping** | 15.645 | [-1867.02 – 1898.31] | 0.987 |
| **Overall** | | | |
|  | **Coef.** | **95% Confidence Interval** | **p value** |
| **Age** | -0.089 | [-0.159 - -0.019] | 0.013 |
| **Gender** | -1.311 | [-3.605 – 0.982] | 0.263 |
| **Duration** | -0.022 | [-0.052 – 0.006] | 0.126 |
| **Type of Mapping** | 15.472 | [-3694.39 – 3725.34] | 0.993 |

**Supplemental Table 3 (*for Table 5*) – Multivariate analysis for the 6 domains of the nTMS-PREMs Questionniare**
